# P-784. Risk Factors for Treatment Failure in Patients with Uncomplicated Urinary Tract Infection

**DOI:** 10.1093/ofid/ofaf695.995

**Published:** 2026-01-11

**Authors:** Steven I Aronin, Sailaja Puttagunta, Jayanti Gupta, Manali Pendse, Michael Dunne

**Affiliations:** Iterum Therapeutics, Old Saybrook, CT; Iterum Therapeutics, Old Saybrook, CT; Iterum Therapeutics, Old Saybrook, CT; Iterum Therapeutics, Old Saybrook, CT

## Abstract

**Background:**

Outpatients presenting with signs and symptoms of uncomplicated urinary tract infection (uUTI) are typically managed with an empiric course of antibiotic therapy. Treatment failure resulting in the need for repeated courses of antibiotics may occur in patients with certain baseline characteristics. In the absence of microbiologic data, typically not available to the prescribing clinician at the time of the clinical encounter, there is a need to define baseline variables predictive of treatment failure in patients with clinical evidence of uUTI. We recently completed two phase 3 studies, SURE-1 and REASSURE, evaluating 3,874 adult women with uUTI.

**Figure:**
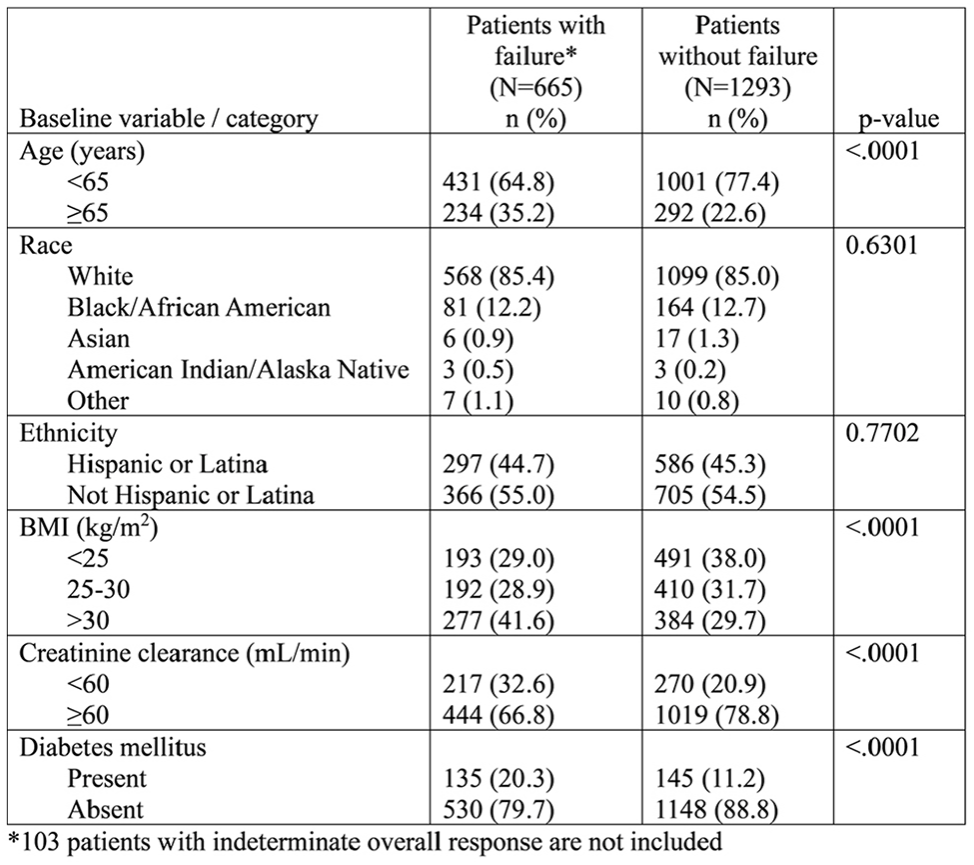
Association Between Baseline Variables and Treatment Outcome

**Methods:**

SURE-1 and REASSURE were randomized, double-blinded, double-dummy, multicenter Phase 3 clinical trials. In the SURE-1 trial, patients with uUTI were randomized to five days of oral sulopenem or three days of ciprofloxacin. In the REASSURE trial, patients with uUTI were randomized to five days of oral sulopenem or five days of amoxicillin/clavulanate. For both trials, the primary endpoint was overall success, defined as both clinical and microbiologic response, at the Day 12/Test of Cure (TOC) visit. Post hoc analyses were performed to determine if there was a relationship between baseline characteristics and overall success for these clinical trial patients.

**Results:**

In the SURE-1 and REASSURE trials combined, there were 2,061 adult female patients with uUTI in the microbiologic Modified Intent to Treat (mMITT) population. The majority of baseline uropathogens recovered were *Escherichia coli* (83.4%) and *Klebsiella pneumoniae* (10.0%). Overall response could be determined for 1,958 (95%) of the patients in the mMITT population. For these clinical trial patients, age > 65 years, BMI > 30 kg/m^2^, creatinine clearance < 60 mL/min and Diabetes mellitus were associated with treatment failure (Table).

**Conclusion:**

Older age, higher BMI, lower creatinine clearance and the presence of Diabetes mellitus identified uUTI patients at increased risk for treatment failure. These variables, plus infection known or suspected to be resistant to one or more commonly prescribed oral antibiotic, may serve to guide clinicians when choosing empiric antibiotic therapy for adult women with uUTI.

**Disclosures:**

Steven I. Aronin, MD, Iterum Therapeutics: Employee|Iterum Therapeutics: Stocks/Bonds (Public Company) Sailaja Puttagunta, MD, Iterum Therapeutics: Stocks/Bonds (Public Company) Michael Dunne, MD, Iterum Therapeutics: Board Member|Iterum Therapeutics: Employee|Iterum Therapeutics: Stocks/Bonds (Public Company)

